# Understanding biological motion through the lens of animate motion processing

**DOI:** 10.3389/fpsyg.2025.1630742

**Published:** 2025-08-12

**Authors:** Li Shen, Xinlin Yang, Yi Jiang, Ying Wang

**Affiliations:** ^1^State Key Laboratory of Cognitive Science and Mental Health, Institute of Psychology, Chinese Academy of Sciences, Beijing, China; ^2^Department of Psychology, University of Chinese Academy of Sciences, Beijing, China

**Keywords:** biological motion, animate motion, animacy perception, life detection, cortical–subcortical network, cross-species

## Abstract

Biological motion (BM), the movement generated by living entities, transmits signals of life and conveys vital cues for animacy perception. In this review, we synthesize empirical findings from human and non-human animal studies to reveal how BM enjoys a unique position in visual perception as an animate motion and how it elicits animacy perception. Compared to non-biological and inanimate motions, BM engages specialized perceptual processing mechanisms and a dedicated cortical–subcortical network. Local motion cues, especially the foot movements of terrestrial animals, are pivotal in driving such specificity, and emerging evidence supports the existence of an innate, evolutionarily conserved “Life Detector” or “Step Detector” tuned to such information in the human and other vertebrate brains. The direct perception of animacy from BM relies on the processing of low-level kinematic features and mid-level motion features embedded in both intrinsic joint movements and extrinsic body motion. While ecological constraints and implied internal energy sources may serve as generic factors affecting animacy perception from visual motion, how precise BM features (both in intrinsic and extrinsic movements) combine to influence animacy percepts and the neural implementation remain largely unexplored. Addressing these gaps will help establish a framework for understanding BM through the lens of animate motion processing. This approach will offer deeper insights into how the life detection system hardwired in the vertebrate brain distinguishes animate from inanimate motion, further uncovering its broader cognitive and evolutionary implications.

## 1 Introduction

Biological motion (BM)—the movement produced by humans or other living creatures—conveys prominent visual cues that elicit a perception of life (Giese and Poggio, 2003; Pavlova, 2012). Compared with static properties of living beings (e.g., forms or surface textures of faces and bodies), BM is more powerful and robust in signaling animacy. It enables rapid detection and efficient perception of animate beings, even under conditions of blurred vision, small object sizes, or great distances (Borst and Egelhaaf, 1989). This ability is essential for survival and reproduction in complex natural environments (Wheatley et al., 2007) and may serve as a gateway to higher-level cognitive processes, such as action understanding and social interaction (Thurman and Lu, 2014; Zanon et al., 2024).

Over the past half-century, numerous studies have revealed the remarkable ability of BM perception in humans (Blake and Shiffrar, 2007), as well as in many other species (Cheng et al., 2025; De Agrò et al., 2021; Jastorff et al., 2012; Larsch and Baier, 2018; Lu et al., 2024; Ma et al., 2022; Nakayasu and Watanabe, 2014; Vallortigara et al., 2005). Human observers could immediately recognize BM from sparse point-light displays that portray only the movement of major body joints (Johansson, 1973). This capacity is inheritable (Wang Y. et al., 2014; Wang et al., 2018), and a visual preference for BM emerges early in life in human infants and visually inexperienced chicks (Bardi et al., 2014, 2011; Simion et al., 2008; Vallortigara et al., 2005). These findings suggest the existence of innate mechanisms, potentially conserved across vertebrates, underlying their superior sensitivity to life motion signals (Lu et al., 2024; Troje and Chang, 2023; Troje and Westhoff, 2006).

**Figure 1:**
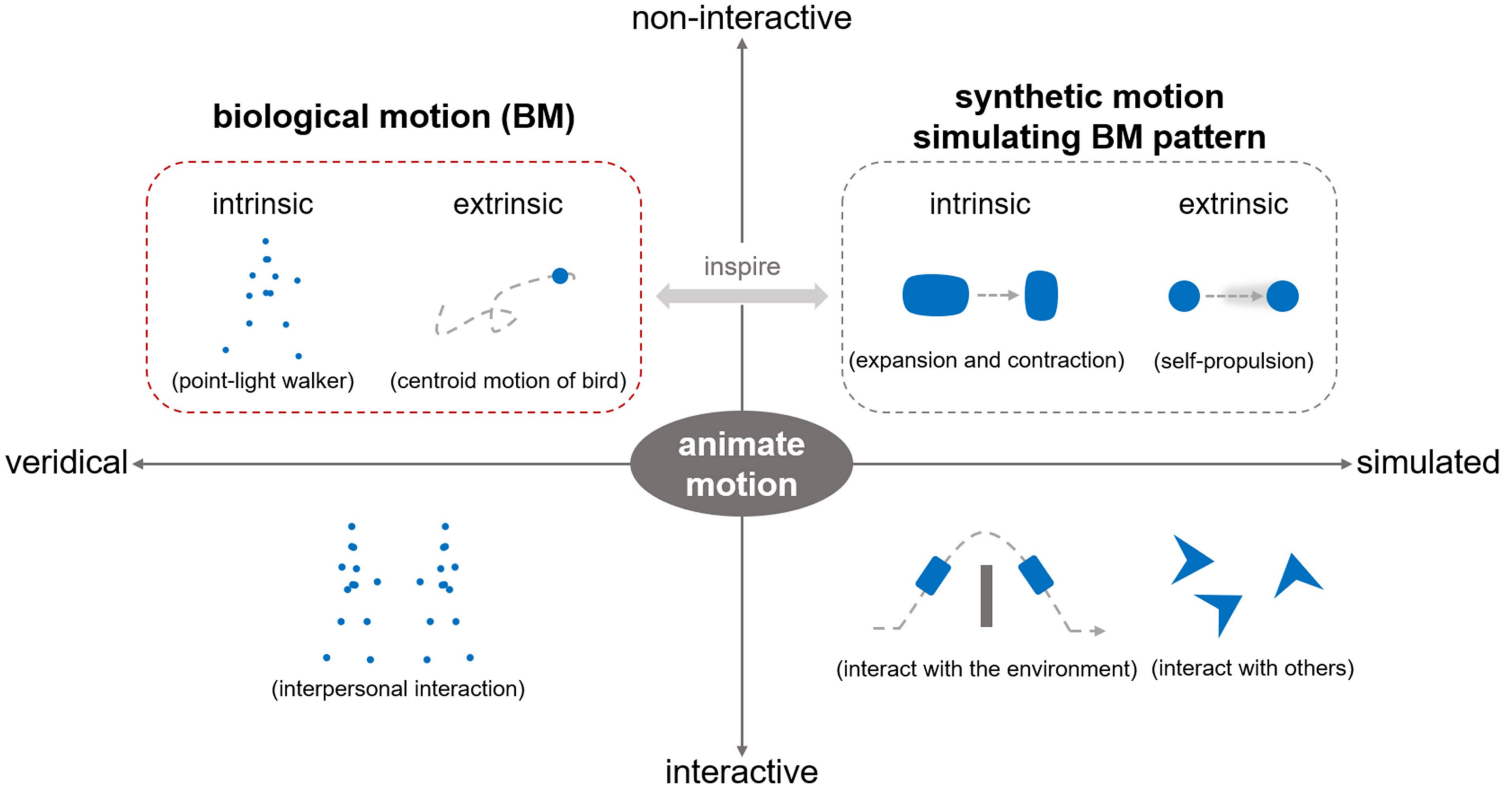
A conceptual framework for decomposing and investigating animate motion. In the literature, the term “animate motion” generally refers to motion that elicits animacy perception. It can be categorized along two dimensions. First, based on engagement with others or the environment, it can be classified as interactive or non-interactive motion. These motion stimuli elicit animate perception via different mechanisms, either through social cognitive inferences or visual feature analysis. Second, based on the motion-generating entity, animate motion can be distinguished as veridical motion or simulated motion. Along this dimension, veridical BM represents the most essential form of animate motion in reality. It consists of intrinsic joint motion and extrinsic body motion components. The area outlined by the red dashed box (i.e., non-interactive BM) represents the primary focus of this review. Additionally, some studies outlined by the gray dashed box also provide insights for understanding animacy perception from BM patterns and are addressed in the third section of the review.

While existing theoretical accounts imply that BM serves as a signal of life that is inherently associated with animacy perception (Chang and Troje, 2008; Johnson, 2006; Troje and Westhoff, 2006), surprisingly few studies have directly explored the mechanisms for the perception of animacy from BM. Animacy perception refers to perceiving an entity as alive or possessing a lifelike quality (Chang and Troje, 2008; Scholl and Tremoulet, 2000; Tremoulet and Feldman, 2000). In the literature, it has been discussed across various contexts, engaging multiple mechanisms at perceptual and cognitive levels (Huang et al., 2023; Rosa Salva et al., 2015). Some research concerns the fast, automatic, and irresistible detection and perception of animate properties from static or motion stimuli (e.g., faces, BM, self-propelled motion) based on visual analysis (Chang and Troje, 2008; Koldewyn et al., 2014; Stewart, 1982). Other studies go further and explore how to infer an entity’s goals and even “mind” based on its interaction with others or the environment (Gao et al., 2010; Heider and Simmel, 1944; Scholl and Tremoulet, 2000), eliciting an impression of an intentional agent (i.e., autonomous and volitional control of action) beyond mere animacy (i.e., being alive, lifelikeness). Here, we focus on animacy perception elicited by non-interactive BM (Figure 1), approaching it primarily from the perspective of visual processing rather than the higher-level mentalizing or social causality comprehension processes. Investigating this issue will expand our understanding of how the human brain processes BM as a signal of life and achieves animacy perception.

In this article, we propose a framework for understanding the neurocognitive architecture of BM perception from the perspective of animate motion processing. We consider BM as the most essential form of animate motion in reality, examining the perceptual and neural mechanisms that underlie (1) its prominent position in visual processing relative to inanimate or less animate motions and (2) its capability to elicit a sense of animacy. We will discuss these topics in the next two sections, while also considering their ontogenetic and phylogenetic origins based on behavioral genetic, early developmental, and cross-species evidence.

Notably, the term BM is sometimes interpreted as the experimental stimulus, namely, point-light displays depicting articulated joint movements. Whereas, this review adopts a broader, ecological perspective, investigating BM as a natural phenomenon—that is, motion produced by living organisms (Giese and Poggio, 2003; Pavlova, 2012). Based on its manifestation, BM can further be decomposed into two components (Figure 1)—intrinsic joint/limb motion (i.e., the relative movements of body parts in the object-based reference system, typically depicted by point-light displays) and extrinsic motion (i.e., movements of the whole body across space) (Thurman and Lu, 2014). Studies on veridical BM sometimes draw inspiration from and inspire those on simulated BM stimuli. Considering the existing research, the second section of this review primarily addresses visual BM processing based on intrinsic motion, and the third section explores animacy perception from BM based on both intrinsic and extrinsic motions.

## 2 Processing BM as animate motion

The significance of BM as a crucial animate motion may have driven the evolution of specialized mechanisms for its perception. In this section, we review empirical findings on the domain-specific perceptual processing mechanisms and neural foundations of BM, as compared to those for inanimate or less animate motions (e.g., random motion, rigid motion, inverted BM). Two fundamental visual cues, namely the local motion capturing the movements of critical joints and the global configuration representing the skeletal structure of bodies, may contribute to these distinctive behavioral and neural responses. We introduce evidence highlighting the crucial contribution of local motion, which leads to theoretical hypotheses about the existence of a cross-species life detection system tuned to local BM cues hardwired in the vertebrate brain.

### 2.1 The specificity of BM perception revealed by human and non-human animal studies

**Figure 2:**
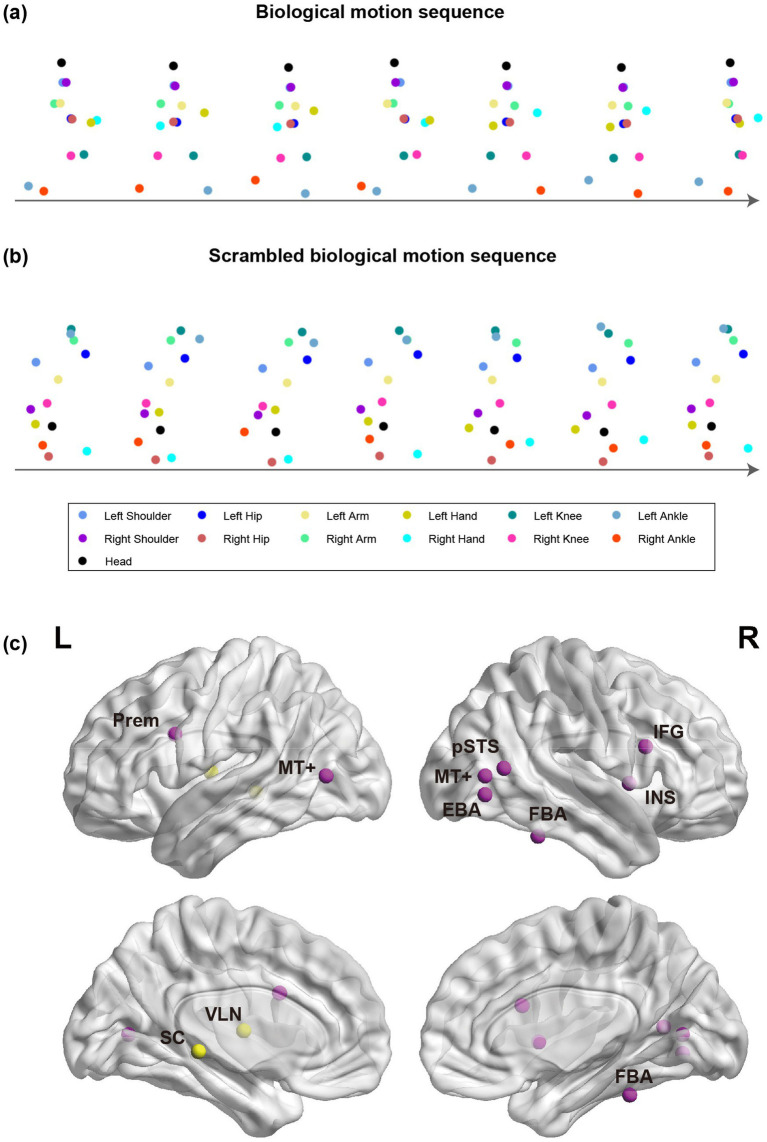
Illustrations of point-light BM sequences and the key brain regions involved in BM processing. **(a,b)** Intact and spatially scrambled BM sequences. **(c)** Cortical (purple) and subcortical (yellow) regions for BM processing in the human brain. SC, superior colliculus; VLN, ventral lateral nucleus; Prem, premotor; pSTS, posterior superior temporal sulcus; MT+, middle temporal complex; FBA, fusiform body area; EBA, extrastriate body area; IFG, inferior frontal gyrus; INS, insula.

Taking advantage of point-light BM displays (Figure 2a), extensive research has revealed the distinctive mechanisms underlying BM perception. Compared with inanimate motions, people recognize BM with higher accuracy and faster speed (Dittrich, 1993). The temporal summation of BM signals occurs over a longer time than that for translational motion and varies with motion speed (Neri et al., 1998). The differences between processing BM and inanimate motion may be traced back to their genetic and evolutionary basis. Twin studies suggest that perceiving the facing direction or motion direction of BM is governed by genetic factors, while the perception of inanimate motions like sphere rotation is largely determined by the environment (Wang Y. et al., 2014; Wang et al., 2018). Furthermore, human newborns spontaneously prefer to look at BM compared to random or rigid motion (Bardi et al., 2011; Simion et al., 2008). This innate preference also extends to other species, including terrestrial vertebrates (e.g., visually inexperienced chick) (Miura and Matsushima, 2012; Vallortigara et al., 2005; Vallortigara and Regolin, 2006) and aquatic vertebrates (e.g., zebrafish) (Larsch and Baier, 2018; Nakayasu and Watanabe, 2014). These findings suggest the existence of a heritable, evolutionarily conserved mechanism selectively dedicated to BM processing.

In addition, the remarkable capability of BM perception is significantly impaired when the visual stimuli are inverted. Compared with inverted counterparts, people demonstrate greater sensitivity in detecting upright BM (Thomas and Shiffrar, 2010) and exhibit enhanced accuracy in discriminating its identity (Loula et al., 2005), walking direction (Troje and Westhoff, 2006), and audiovisual temporal relations (Saygin et al., 2008). Moreover, for both human infants and visually inexperienced chicks, there is an innate preference for upright BM over the inverted counterparts (Bardi et al., 2014; Vallortigara and Regolin, 2006). Since inversion disrupts gravity-compatible motion cues but not the low-level properties of upright BM, this cross-species disposition is considered a “gravity bias” that reflects the visual system’s selective tuning to life motion signals within the Earth’s gravitational field. This bias may, to some extent, contribute to the advantage of gravity-compatible BM in visual perception, presumably reflecting the adaptation of terrestrial life to Earth’s environment. Consistent with this assumption, a recent study has demonstrated that exposure to microgravity during spaceflight significantly reduced the inversion effect in BM perception, suggesting the significant role of the gravitational environment in shaping the visual sensitivity to BM (Wang et al., 2022).

### 2.2 The significance of local motion cues in life motion perception

BM perception relies on the processing of global configuration and local motion cues (Chang and Troje, 2009b). While early studies emphasized the significance of global configuration processing (Beintema and Lappe, 2002; Bertenthal and Pinto, 1994; Dittrich, 1993; Lange and Lappe, 2006; Reed et al., 2003; Shiffrar et al., 1997), later work by Troje and Westhoff proposed that local motion processing is another independent component of BM perception (Troje and Westhoff, 2006). Local motion cues can be extracted by spatially scrambling the point-light BM stimuli (Figure 2b), which disrupts the global configuration but preserves the movements of each joint. Troje and Westhoff found that humans can retrieve walking direction information from scrambled BM, whereas inverting the scrambled stimuli severely impaired visual perception, inducing an inversion effect comparable to that obtained with intact BM. Such an inversion effect is mainly driven by the motions of the feet, as inverting only the feet has a much stronger impact on walking direction discrimination compared to inverting all points except the feet (Troje and Westhoff, 2006). The following research suggests that local BM alone can modulate a variety of cognitive processes (e.g., visual attention, visual awareness, time perception, audiovisual integration) at levels comparable to those elicited by intact BM (Shen et al., 2023a; Shen et al., 2025a; Shi et al., 2010; Sun et al., 2022; Wang and Jiang, 2012; Wang L. et al., 2014). Moreover, both human newborns and newly hatched chicks exhibit comparable visual preferences toward intact BMs and scrambled ones (Bardi et al., 2011; Vallortigara et al., 2005), suggesting that the cross-species visual preference for BM may be primarily driven by local motion cues, even though the global configuration can modulate such effects (Bardi et al., 2014; Hirai et al., 2011). This is likely because local motion cues convey gravitational acceleration and exhibit semi-rigid characteristics, both of which have been implicated in eliciting spontaneous attention preferences (Bardi et al., 2011; Vallortigara and Regolin, 2006).

### 2.3 Theoretical accounts of life motion perception

The above findings have provided converging support for the “life detector” hypothesis originally proposed by Troje and Westhoff, which posits the existence of a specialized system in the human brain, and potentially in other vertebrates, that enables efficient detection of life motion signals based on distinctive local motion cues (Lemaire and Vallortigara, 2022; Troje and Chang, 2023; Troje and Westhoff, 2006). Recently, Hirai and Senju (2020) proposed a two-process theoretical model for the development of BM perception, parallel with the two-process model of face and gaze processing (Johnson et al., 2015; Senju and Johnson, 2009). According to this model, BM processing engages two systems: the Step Detector and the Bodily Action Evaluator. The Step Detector system quickly and coarsely processes the local feet motion and the feet-below-body information. It detects life motion signals generated by both conspecifics and other vertebrates and is largely innate. The Bodily Action Evaluator system slowly and precisely processes the global structure-from-motion information. It selectively responds to the motion from conspecifics and is mainly shaped by learning. The two-process model systematically develops and extends the earlier “Life Detector” hypothesis. The Step Detector system is generally compatible with the original Life Detector hypothesis but highlights the significance of feet-related motion and configurational information. The Bodily Action Evaluator engages in global configuration processing that was underrepresented in the Life Detector framework. The two-process model also characterizes the different developmental trajectories for the two systems, which are consistent with findings from a twin study that local and global BM perception are determined, respectively, by genetic and environmental factors (Wang et al., 2018). Based on the characteristics of the two systems, Hirai and Senju further hypothesized that the two systems involve distinct neural substrates, with the first system primarily relying on subcortical processing and the second system relying on cortical processing. In the next section, we will review the relevant evidence.

### 2.4 A cortical–subcortical network for BM processing

Previous reviews have provided comprehensive summaries of the human cortical network engaged in BM perception (Blake and Shiffrar, 2007; Giese and Poggio, 2003; Hirai and Senju, 2020). Figure 2c provides a brief overview of these cortical regions, including the superior temporal sulcus (STS), extrastriate body area (EBA), fusiform body area (FBA), middle temporal area (MT+), premotor cortex, inferior frontal gyrus (IFG), and insula. The STS is a core region that integrates form and motion cues to construct coherent representations of BM (Jastorff and Orban, 2009; Sokolov et al., 2018). Beyond human studies, a few non-human animal studies and cross-species studies offer insights into the evolution of this cortical network. By contrasting the neural response to BM versus that to static figures or non-BM, previous research has demonstrated STS activations in both monkeys and humans, regardless of whether the motion is generated by conspecifics or nonconspecifics, although with stronger responses to conspecific motions (Jastorff et al., 2012). By comparing the neural response to BM with that to inverted stimuli, a recent study found a selective response to conspecific motion in the human STS but not in the monkey STS, while the MT region in human as well as monkey brains responds to both conspecific and nonconspecific motions without conspecific preference (Cheng et al., 2025). Taken together, these findings indicate that upstream cortical regions (i.e., MT) may retain homologous functions across species (i.e., lack conspecific selectivity), and downstream cortical regions (i.e., STS) may have undergone differentiation and specialization throughout evolution.

Recent studies have also explored the subcortical processing of BM (Figure 2c). One study found that the ventral lateral nucleus (VLN) is involved in the processing of both global configuration and local motion cues of BM (Chang et al., 2018). Another recent study based on 3 T and 7 T functional magnetic resonance imaging (fMRI) found that the superior colliculus (SC), a subcortical structure involved in early visual processing, is activated more strongly by upright scrambled BM compared to the inverted counterpart (Lu et al., 2024). Moreover, it reveals a subcortical–cortical functional pathway from SC through the MT to the pSTS in the human brain. More potential subcortical–cortical connectivities remain to be examined in the future. The activation of SC is observed not only in humans but also in macaque monkeys, suggesting a cross-species mechanism in the primate SC that facilitates the detection of local BM at the early stage of the visual processing stream. The subcortical encoding of BM has also been observed in non-primates. The preoptic area and septum of newly hatched chicks are involved in discriminating BM from rigid motion (Lorenzi et al., 2024; Mayer et al., 2017). The visual input to the preoptic area in chicks is mainly provided by the optic tectum, an area homologous to the mammalian SC and responsible for the detection of animate cues, especially dynamic motion cues (Rosa Salva et al., 2015). These findings suggest that the processing of local motion cues may recruit early and primitive brain areas in different species (e.g., SC, preoptic area), providing supporting evidence for the existence of a “Step Detector” (Hirai and Senju, 2020) or a “life detector” (Troje and Westhoff, 2006) system in the evolutionarily ancient subcortical structures.

In addition to the cerebrum, several neuroimaging studies have reported cerebellum activation during BM processing (Sokolov et al., 2012, 2018), although one lesion study found that cerebellum dysfunction did not impair the detection of BM (Jokisch et al., 2005). Furthermore, BM perception may also rely on embodied motor simulation (Arrighi et al., 2011; Bosbach et al., 2006; Cavallo et al., 2012; Wilson and Knoblich, 2005). By modulating the availability of online stimulation, a recent study suggests that sensorimotor simulation at peripheral effectors plays an irreplaceable role in processing BM, especially for local motion cues (Shen et al., 2025a). The brain–body entanglement mechanism in BM perception may provide a certain physiological basis for the timely execution of fight-or-flight and other action responses.

## 3 Animacy perception from BM

Although BM is a vital signal for the existence of life, how it leads to animacy perception remains largely unknown. In the current section, we review empirical evidence on this issue. Compared to other attributes of BM, animacy is a more fundamental and vital dimension associated with life, given its invariance across different circumstances and its role as a prerequisite for conveying other attributes (e.g., emotion) unique to living entities. Note that the perception of animacy and other attributes from BM is not perceptually independent. Chang and Troje (2008) found a significant correlation between walking direction discrimination and animacy ratings for point-light walking motion, suggesting potentially shared visual mechanisms underlying the two processes, such as visual motion analysis. However, perceiving animacy from BM should not be conflated with the processing of walking directions, as these processes serve distinct perceptual and cognitive functions. Walking direction conveys others’ goals and interests in the surroundings. Thus, perceiving and discerning walking direction enables rapid access to the direction of others’ intentions and automatic orienting toward it through a specialized social attentional mechanism (Ji et al., 2020; Shi et al., 2010; Wang L. et al., 2014; Wang et al., 2020; Zhao et al., 2014). In comparison, animacy perception refers to perceiving an entity as alive or possessing agency (Scholl and Tremoulet, 2000; Vallortigara, 2012). It enables organisms to differentiate between animate and inanimate objects and develop appropriate adaptive responses.

As previously mentioned, this article focuses on animacy perception elicited by non-interactive BM, exploring the mechanism for perceiving animacy based on visual motion processing rather than social cognitive inferences. We will introduce key factors and motion features that influence animacy perception based on intrinsic and extrinsic motion patterns, respectively, and explore the potential neural substrates.

### 3.1 Perceiving animacy from the intrinsic joint movements of BM

Only a few studies explored how intrinsic joint movements of BM lead to animacy perception. Most of them utilized walking stimuli, one of the most prevalent types of locomotion for terrestrial vertebrates, based on rating tasks or comparing visual stimuli on the animacy dimension. By measuring the animacy perception induced by point-light sequences of a walking human, cat, and pigeon, Chang and Troje (2008) found that upright stimuli were rated more animate than inverted stimuli. Such inversion effect of animacy rating also existed in scrambled point-light BM sequences, in which the local trajectories are preserved while the global configurations are disrupted. It suggests that at least part of animacy information is carried by the local motion cues without relying on global configuration. In addition, since the inverted point-light displays alter only the motion in the vertical direction while preserving the horizontal component, the inversion effect may indicate the influence of gravity direction on the animacy judgment of local motion. Thurman and Lu (2013) further found that gravity direction and the congruency between intrinsic and extrinsic motion have an interactive influence on animacy percept. Animacy ratings only increased for spatially scrambled point-light walkers with upright orientation and congruent intrinsic-extrinsic motion direction, compared with inverted or incongruent conditions. Taken together, these findings underscore the importance of local BM with ecological constraints (both physical and biological) in perceiving animacy. Combined with prior evidence for the influence of the gravitational environment on BM processing (Wang et al., 2022), these findings suggest that the ecological environment, along with the concomitant constraints and expectations, may shape the perception of animate motion information.

### 3.2 Perceiving animacy from extrinsic motion patterns

In addition to the intrinsic joint movement of animate agents, extrinsic motion patterns also constitute an integral component of BM and embody vital life signatures. While most studies on BM perception adopted point-light displays that eliminate extrinsic body locomotion, some evidence suggests that the extrinsic motion of BM also modulates its perception and animacy rating (Masselink and Lappe, 2015; Thurman and Lu, 2013). Even without intrinsic relative motion, observers can easily discriminate animate motion from inanimate one in the real world based on their low-level kinematics and trajectories of extrinsic motion (Han et al., 2023). Given the complexity of real animate motion, some researchers used a single inanimate object without animate form or texture (e.g., a dot or a geometrical shape) to simulate extrinsic motions, allowing the isolation of key motion factors that contribute to animacy impression under rigorous experimental control. Although these simulated motions are not identical to veridical BMs, identifying the critical motion factors in such stimuli that trigger animacy perception could offer insights into how we perceive animacy from the extrinsic motion patterns of living creatures.

A large number of studies have demonstrated that self-propelled motion elicited a stable perception of animacy. Observers attributed animacy to geometric shapes exhibiting autonomous motion initiation (Stewart, 1982), speed modulation, direction change, or alignment between the principal axis orientation and its motion direction (Tremoulet and Feldman, 2000). Another cue signaling animacy is motion against gravity. Szego and Rutherford (2008) demonstrated that extrinsic motions inconsistent with gravity elicited stronger animacy perception than those consistent with gravity. In particular, more participants judged the upward motions (against gravity) of a single dot as more animate compared with the downward ones presented on a vertically oriented computer screen. However, when the screen was horizontal (i.e., parallel to the ground), altering the directional relationship of the motion and the gravitational context, participants no longer showed preference in their animacy judgments. This gravitational modulation of animacy judgments suggests that gravity is an ecological constraint shaping animacy perception from extrinsic motion, and identifying motion against the gravitational force may be essential for animacy perception.

Although these findings were derived from the extrinsic motions along synthetic paths, terrestrial, aquatic, and aerial animals in the natural world are all able to initiate motion autonomously and move against gravity, driven by an internal energy source. Cross-species evidence suggests that the visual sensitivity to such motion patterns emerges early in life. Di Giorgio et al. (2017) first demonstrated human newborns’ ability to differentiate between self- and non-self-propelled objects and their visual preference toward self-produced motion. Studies on visually-naïve chicks demonstrated their spontaneous preference for a geometric shape’s motion exhibiting speed changes (Rosa-Salva et al., 2016), axis-path parallelism, or spontaneous rotation (Rosa-Salva et al., 2018), relative to constant-speed, non-parallel, or translational motions. Moreover, newborn chicks have a predisposition to approach stimuli moving against gravity (Bliss et al., 2023). These findings, together with the abovementioned evidence, open the possibility that identifying internal energy sources indicated by particular motion features (i.e., self-propelled, against-gravity) serves as a general mechanism for animacy perception from visual motion. They also suggest an early ontogenetic origin of the capacity to detect animate motion and perceive animacy based on some general animate kinematic features, possibly driven by a phylogenetically old mechanism shared among various vertebrates.

### 3.3 Comparison of animacy perception between intrinsic and extrinsic motions

Beyond those general factors, cognitive processing mechanisms for intrinsic and extrinsic motion still differ in some aspects. For instance, the perception of extrinsic motion does not require the complex analysis and integration of intrinsic kinematic characteristics. This may account for the seemly contradictory findings that the gravity-incompatible intrinsic movements in inverted point-light BM reduce animacy perception while the extrinsic motion of a single point against gravity enhances animacy perception. Since the local kinematics of intrinsic joint ballistic movements reflect the combined influences of gravity and biomechanics, the inverted gravity-incongruent movements impair this inherent ecological validity and thus reduce perceived animacy. In comparison, the downward global extrinsic motion indicates only the gravitational constraint, therefore the ability to move against gravity can be interpreted as evidence of an internal energy source generated by an animate agent, prompting observers to perceive animacy.

While these findings can be understood within a unified framework considering fundamental physical and biological constraints, the similarities and differences in animacy impression mediated by intrinsic versus extrinsic motions and their integration remain underexplored. Thus, further investigation is required to advance our understanding of animacy perception from BM by considering both intrinsic and extrinsic motions.

### 3.4 Neural mechanism for animacy perception from motion cues

Most studies on the neural mechanism of point-light BM focused on its visual processing rather than the animacy percepts. Among the studies concerning animacy perception, most elicited higher-level social cognitive processes along with basic animacy impressions by imposing inter-object or object-environment interactions to simple objects. Only a limited number of studies have preliminarily investigated the neural substrates for animacy perception based on the visual processing of motion cues, and these studies typically examined the extrinsic motions of simple, non-interactive objects.

Schultz and Bülthoff (2019) have demonstrated that the intraparietal sulcus (IPS) serves as a key cortical region for perceiving animacy from extrinsic motion patterns. Using simulated animation sequences showing a single dot’s self-propelled motion transitioning through six morph levels from inanimate to animate, they found that the right IPS showed a higher response when observers perceived animacy compared with when they did not. However, they did not observe the involvement of some typical cortical regions associated with BM perception, such as the pSTS. This discrepancy may occur because perceiving animacy from such a simplified moving stimulus does not rely on form-motion integration or action recognition. It is likely that animacy perception from different motion cues, particularly the intrinsic joint movements in point-light displays and the global extrinsic motion of a single dot, involves distinct neural substrates. Therefore, it is necessary to reassess the functional contributions of brain regions engaged in complex BM processing and elucidate their role in perceiving animacy from motion cues.

Moreover, evidence from studies in humans and other animals indicates the role of subcortical structures in perceiving general animate motion with different forms. Previous studies have demonstrated that chicks exhibit innate predispositions for animate motion eliciting animacy perception in humans (Bliss et al., 2023; Rosa-Salva et al., 2016, 2018; Vallortigara et al., 2005; Vallortigara and Regolin, 2006). Given the substantial brain organization differences and phylogenetic distance between species, this cross-species mechanism potentially involves evolutionarily ancient neural substrates (Hirai and Senju, 2020; Johnson, 2006; Troje and Westhoff, 2006). More specifically, certain homologous subcortical structures may contribute to the cross-species capacity for innate and rapid perception of animacy, distinct from cortical elaboration. Lorenzi et al. (2017) found that the right septum and the preoptic area of newly hatched chicks exhibited higher neuronal activity when they were exposed to the motion of a disk with speed changes compared to that at a constant speed. These findings, combined with the recent empirical results of the preoptic area involvement in BM perception in newborn chicks (Lorenzi et al., 2024), establish evidence for the crucial role of the preoptic area, a conserved subcortical structure, in general animate motion processing at birth. In addition, the SC is a pivotal subcortical region for processing local motion cues of BM in humans and macaques (Lu et al., 2024), as previously discussed. Given the contributions of local motion in perceiving animacy (Chang and Troje, 2008; Thurman and Lu, 2013), not just in processing BM, the SC may also be closely related to animacy detection and perception in primates. However, since these findings are based on the visual processing of animate versus inanimate motion rather than being directly linked to animacy percept, the exact role of subcortical structures in perceiving animacy from motion cues remains to be delineated.

## 4 Discussion and future directions

In this review, we focus on the perceptual and neural processing of a vital type of animate motion, namely, BM, spanning from its visual perception to animacy perception based on visual motion analysis. By reviewing key findings in human and non-human animal studies, we have outlined the specialized processing of BM across different species and emphasized the role of local motion cues in driving the domain-specific mechanisms. Compared to the emerging understanding of the cortical–subcortical networks involved in BM processing, much less is known about the neural networks underlying animacy perception from BM. The latter topic requires more systematic and in-depth investigations.

In addition, what key motion features in BM elicit animacy perception remains to be investigated. The current “Life Detector” and “Step Detector” hypotheses emphasize the significance of low-level kinematic features, namely the acceleration profile of local BM and especially the foot motions, in perceiving walking direction as well as the animate properties of a creature (Chang et al., 2018; Chang and Troje, 2008, 2009a; Hirai and Senju, 2020; Johnson, 2006; Troje and Westhoff, 2006). Between processing low-level local motion and perceiving global BM, mid-level motion features provide structured motion units that capture spatiotemporal relations between joints. Previous research employing simulated motion stimuli suggests that some mid-level motion features, such as the combination of deformation and translation (Kawabe, 2017), as well as alternating contraction and expansion accompanied by translational movement (Parovel et al., 2018), could trigger the impression of animacy. Other mid-level features, such as opponent motion, are crucial for BM perception (Casile and Giese, 2005; Thurman and Grossman, 2008), while their role in animacy perception remains unclear. In addition, how low-level kinematic features and mid-level motion patterns are integrated to support animacy perception and agency attribution also awaits further investigation. Exploring these questions may not only help broaden our understanding of how the brain identifies BM as a signal of life but may also provide valuable insights for designing bio-robots capable of producing life-like movements.

Notably, rhythm is an inherent and intrinsic feature of basic locomotion patterns across many species, such as the walking of humans, the swimming of jellyfish, and the flying of butterflies. Moreover, like BM processing, rhythm processing also has an evolutionary basis (Kotz et al., 2018). These suggest a potential link between rhythm processing and animacy perception elicited by BM. Previous research has shown that neural oscillations will align to and continuously track the temporal structures in external and internal rhythms (Lakatos et al., 2019; Thut et al., 2011), a mechanism that enables humans to maintain perceptual and cognitive representations of rhythmic stimuli even after they have disappeared (Miller et al., 2013). Recent electroencephalograph (EEG) studies have found that the neural tracking of rhythmic structures in human walking movements contributes to domain-specific encoding of BM signals (Shen et al., 2023b; Shen et al., 2025b). These findings raise an important question: does the neural encoding of locomotion rhythms also contribute to the animacy perception elicited by BM?

Finally, future studies could integrate the investigation of neural mechanisms underlying animacy perception from visual motion cues with that from static cues. Previous work has revealed a lateral-to-medial functional gradient organization in the ventral visual pathway for distinguishing animate from inanimate categories through static cues (Grill-Spector and Weiner, 2014; Kriegeskorte et al., 2008). Apart from the ventral temporal cortex (VTC), the superior temporal sulcus (STS) is also sensitive to static animate cues such as face and body (Allison et al., 2000; Pinsk et al., 2009). Static animate images and dynamic animate cues seem to share a part of overlapping neural substrates, including the STS, despite differences in visual representation. However, it remains unclear whether static and motion cues elicit distinctive activation patterns in these regions, how integration occurs between the processing of static and motion cues, and whether they rely on a common core network or dissociable pathways in evoking animacy perception. Addressing these questions would lead to a more comprehensive understanding of the neurocognitive mechanism of animacy perception.
